# Intrinsic correlation between β-relaxation and spatial heterogeneity in a metallic glass

**DOI:** 10.1038/ncomms11516

**Published:** 2016-05-09

**Authors:** F. Zhu, H. K. Nguyen, S. X. Song, Daisman P. B. Aji, A. Hirata, H. Wang, K. Nakajima, M. W. Chen

**Affiliations:** 1 State Key Laboratory of Metal Matrix Composites, School of Materials Science and Engineering, Shanghai Jiao Tong University, Shanghai 200030, China; 2 WPI Advanced Institute for Materials Research, Tohoku University, Sendai 980-8578, Japan

## Abstract

β-relaxation has long been attributed to localized motion of constituent molecules or atoms confined to isolated regions in glasses. However, direct experimental evidence to support this spatially heterogeneous scenario is still missing. Here we report the evolution of nanoscale structural heterogeneity in a metallic glass during β-relaxation by utilizing amplitude-modulation dynamic atomic force microscopy. The successive degeneration of heterogeneity during β-relaxation can be well described by the Kohlrausch–Williams–Watts equation. The characteristic relaxation time and activation energy of the heterogeneity evolution are in accord with those of excess enthalpy release by β-relaxation. Our study correlates β-relaxation with nanoscale spatial heterogeneity and provides direct evidence on the structural origins of β-relaxation in metallic glasses.

## Abstract (web summary)

Beta-relaxation in glasses is commonly attributed to the confined motions of constituent atoms in nanosized domains, but there is no direct evidence so far. Here, Zhu *et al*. show the correlation between the evolution of spatial heterogeneity at nanoscale and beta-relaxation below glass transition point.

## Graphical abstract

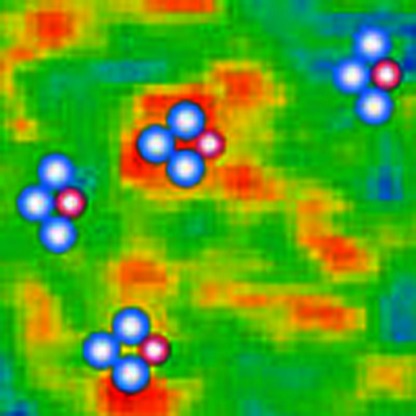

When a glass-forming liquid is cooled down to the moderately supercooled regime, the high-temperature single-relaxation process splits into α- and β-relaxations^1^,^2^,^3^,^4^,^5^. The α-relaxation is related to glass transition and vanishes at a glass transition temperature (*T*_g_), whereas the β-relaxation or Johari–Goldstein relaxation remains as the principal source of dynamics in glasses^4^,^5^,^6^. Recent studies have demonstrated that the β-relaxation plays important roles in structural relaxation^7^, accelerated partial devitrification^8^ and deformation of metallic glasses^9^,^10^, showing both scientific and practical significances in glass dynamics. However, microscopic origins of β-relaxation have not been fully understood and theoretical models remain evolving^2^,^3^,^4^,^5^,^6^,^7^,^8^,^9^,^10^,^11^,^12^,^13^,^14^,^15^,^16^,^17^. It has been originally suggested by Johari^11^ that the β-relaxation in mechanically rigid glasses, especially in metallic glasses, should be processed by the translational motion of atoms localized in loosely packed regions. This scheme certainly assumes that β-relaxation intrinsically correlates with and structurally originates from structural heterogeneity of glasses. Although structural heterogeneity has been observed in metallic glasses at different length scales by experiments^8^,^18^,^19^,^20^,^21^,^22^,^23^,^24^,^25^,^26^ and simulations^27^,^28^,^29^,^30^,^31^, the direct evidence on the intrinsic correlation between structural heterogeneity and β-relaxation in metallic glasses is still missing. Consequently, the structural origins of β-relaxation are still under debate despite many efforts devoted to this long-standing problem.

In this study, we utilize amplitude-modulation dynamic atomic force microscopy (AM-AFM)^20^,^32^,^33^ to characterize the successive changes of structural heterogeneity in a metallic glass regulated by the sub-*T*_g_ enthalpy relaxation. It is found that the characteristic relaxation time and activation energy of the heterogeneity dynamics are approximately equal to those of β-relaxation measured by excess enthalpy release using a hyper-quenching–annealing–calorimetric scan approach^7^. The quantitative measurements of the dynamics for both spatial heterogeneity and β-relaxation in one system provide direct evidence on the intrinsic correlation between β-relaxation and heterogeneous structure in metallic glasses, which reveals the structural origin of sub-*T*_g_ relaxation of metallic glasses.

## Results

### Sub-*T*_g_ relaxation

**Figure 1:**
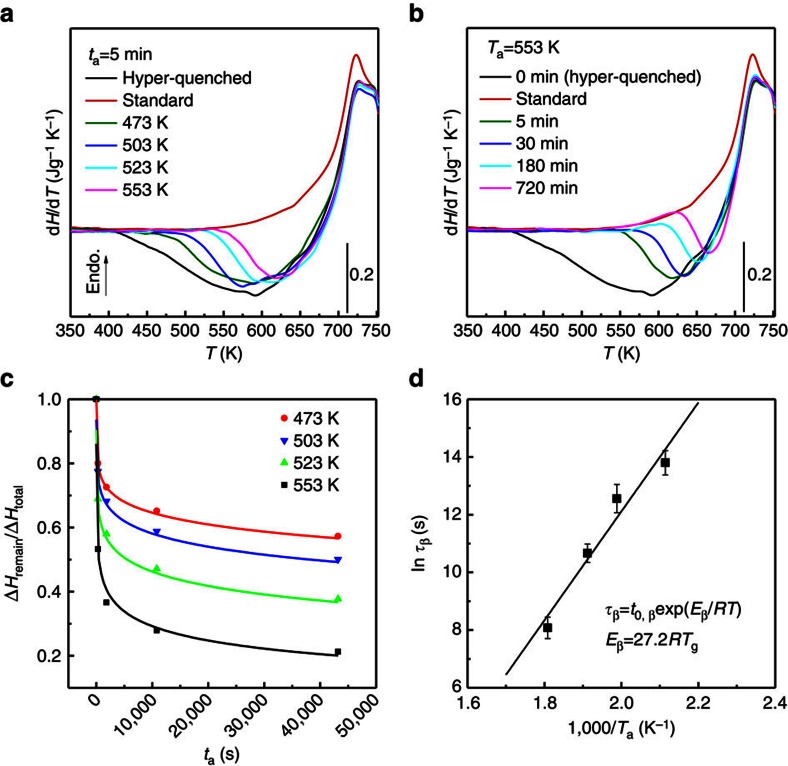
Structural relaxation of hyper-quenched metallic glass below *T*_g_. (**a**) Heat flow traces of the glass samples relaxed at *T*_a_=553 K for different durations; and (**b**) the samples annealed at different temperatures *T*_a_ for *t*_a_=5 min. (**c**) Annealing time *t*_a_ dependence of the normalized remaining enthalpy for the samples annealed at 473, 503, 523 and 553 K and their corresponding fittings by KWW function. (**d**) Dependence of the characteristic relaxation time *τ*_β_ on the reciprocal annealing temperature (1/*T*_a_), surrendering the activation energy of sub-*T*_g_ relaxation. The error bars indicate standard deviation. Endo., endothermic.

To successively change the local structure of the hyper-quenched metallic glass, the glass samples are relaxed by annealing at sub-*T*_g_ temperatures from *T*_a_=473 K (∼0.68*T*_g_, *T*_g_=695 K) to 553 K (∼0.8*T*_g_) for different durations from *t*_a_=5 min to 720 min. For reference, the hyper-quenched metallic glass without annealing is marked with *t*_a_=0 min. Figure 1 shows the differential scanning calorimeter (DSC) profiles of the sub-*T*_g_ relaxation. Compared with the standard sample, the hyper-quenched sample exhibits a broad exothermic peak below *T*_g_, indicative of the abundant excess enthalpy trapped in the glass with a structure far away from equilibrium^16^. At *T*_a_=553 K, the excess enthalpy is sequentially released with annealing time (Fig. 1a). At a constant annealing time *t*_a_=5 min, more excess enthalpy is released at higher *T*_a_ (Fig. 1b). As demonstrated by the previous DSC measurements^7^,^16^, the β-relaxation appears as the tails at the low-temperature side of the broad sub-*T*_g_ exothermic peaks in the DSC traces. Our enthalpy relaxation is controlled below 0.8*T*_g_ at which the sub-*T*_g_ relaxation shows irrelevant influence on the glass transition as the DSC traces above *T*_g_ are almost identical before and after annealing (see Fig. 1a and Supplementary Fig. 1). Thus, it can be concluded that the sub-*T*_g_ relaxation below 0.8*T*_g_ is mainly processed by the β-relaxation. The remaining excess enthalpy Δ*H*_remain_ for the samples annealed below 553 K is normalized by the total excess enthalpy Δ*H*_total_ measured from the hyper-quenched sample and plotted against *t*_a_ as shown in Fig. 1c. The time dependence of the normalized enthalpy release at each annealing temperature can be well fitted by the Kohlrausch–Williams–Watts (KWW) function: 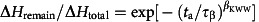, where *τ*_β_ is the characteristic relaxation time and *β*_KWW_ is the stretch parameter for the fitting. The KWW function is often used to describe the temporal behaviour of disordered systems in response to external stimulus, such as high temperature, applied electric field, stress and imposed deformation^1^. The good fitting of the normalized sub-*T*_g_ enthalpy release confirms the previous suggestion that the KWW function is appropriate for time-related processes of β-relaxation^1^,^7^. Since the β-relaxation also obeys the Arrhenius law^1^,^7^, the temperature dependence of the relaxation time, *τ*_β_, derived from the KWW fittings, follows the Arrhenius equation: 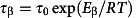. The activation energy *E*_β_ for the sub-*T*_g_ relaxation can be obtained by plotting the logarithm of the relaxation time *τ*_β_ versus the reciprocal annealing temperature (1/*T*_a_). The linear relationship in Fig. 1d gives a value of *E*_β_ around (27.2±4.3)*RT*_g_, which is in accord with the empirical value *E*_β_=∼26*RT*_g_ of β-relaxation in metallic glasses, polymers, rigid molecules and other glasses, but much smaller than that of α-relaxation^4^,^6^,^7^,^16^,^34^. Particularly, this value is consistent with the activation energy for β-relaxation of rapidly quenched metallic glasses in previous thermal measurements^7^,^16^ and dynamic mechanical analyses^35^. Therefore, the β-relaxation dominates the excess enthalpy release and the structural changes during the sub-*T*_g_ relaxation of the hyper-quenched metallic glass.

**Figure 2:**
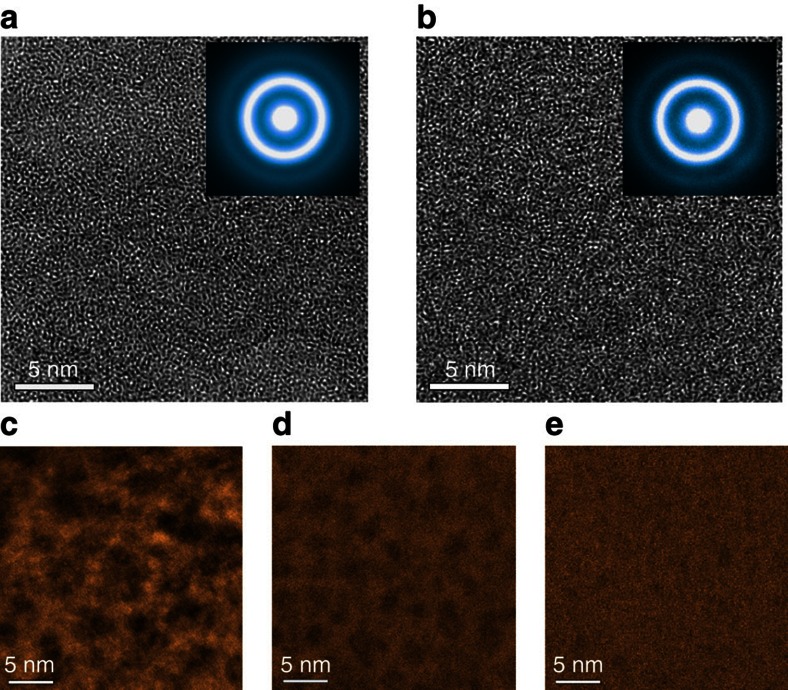
Microstructure of the hyper-quenched and sub-*T*_g_ relaxed metallic glass. HRTEM images of (**a**) the hyper-quenched metallic glass and (**b**) the metallic glass relaxed at 553 K for 720 min. High-angle annular dark-field scanning TEM (HAADF-STEM) images of (**c**) the hyper-quenched metallic glass, (**d**) the metallic glass relaxed at 553 K for 5 min and (**e**) relaxed at 553 K for 720 min. All the samples were prepared using gentle ion milling.

The glass samples before and after the annealing are inspected by X-ray diffraction (Supplementary Fig. 2), X-ray energy dispersive spectroscopy (Supplementary Fig. 3) and X-ray photoelectron spectroscopy (XPS) (Supplementary Fig. 4). No evident changes in amorphous structure and chemistry, particularly oxygen concentration, can be seen, suggesting that the annealing does not cause detectable crystallization or oxidation. The detected oxygen in the X-ray photoelectron spectroscopic spectra is mostly from the surface adsorption of carbonates, as well as from the topmost surface oxidation after the samples are exposed to an atmosphere. Phase-contrast high-resolution transmission electron microscopy (TEM) images show the homogeneously amorphous structures of the hyper-quenched (Fig. 2a) and relaxed samples (Fig. 2b). The corresponding diffraction halos (insets in Fig. 2a,b) confirm their amorphous natures. However, obvious contrast variation can be observed in the hyper-quenched metallic glass by high-angle annular dark-field scanning TEM (Fig. 2c). The size of dark regions, corresponding to low density domains, is ranging from 5 to 7 nm. After the sub-*T*_g_ relaxation at 553 K, the dark regions become weaker and smaller with the increase of annealing time (Fig. 2d,e). Since all TEM samples are prepared by ion milling with the same conditions, such contrast difference may be associated with the nanoscale structural heterogeneity of metallic glass. More detailed TEM analysis of the inhomogeneous structure in metallic glasses will be reported elsewhere.

### Amplitude-modulation AFM

**Figure 3:**
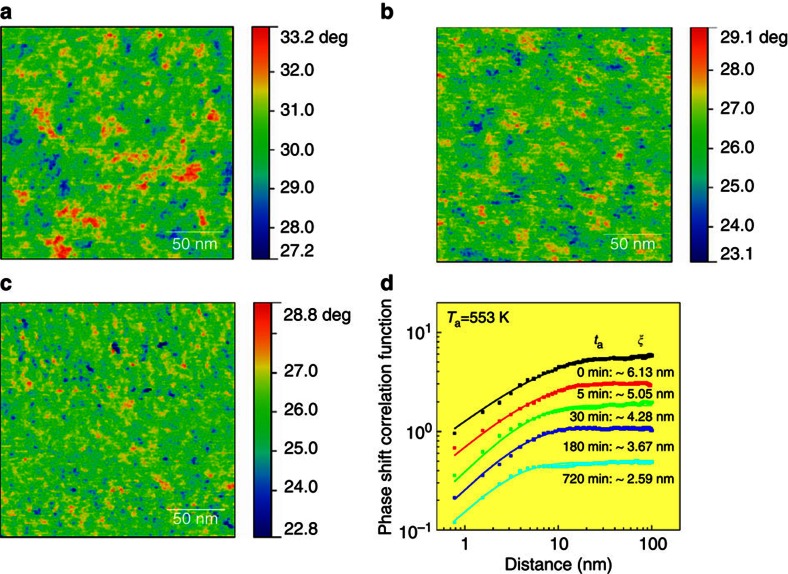
Evolution of spatial heterogeneity during sub-*T*_g_ relaxation at *T*_a_=553 K. Phase shift images of (**a**) the hyper-quenched metallic glass, (**b**) the metallic glass relaxed at 553 K for 5 min and (**c**) relaxed at 553 K for 720 min. (**d**) Correlation function curves of the samples annealed at 553 K for different durations. The correlation lengths of spatial heterogeneity in phase shift images can be determined by the correlation function curves. Note that the curves were shifted vertically for clear identification.

Figure 3a presents the AM-AFM phase shift image of the hyper-quenched metallic glass before and after the sub-*T*_g_ relaxation at 553 K. Since the phase lags in the AM-AFM image arise from the energy dissipation during tip–sample interactions, the viscoelastic origin of the phase shift can be determined by measuring the normalized dissipated energy versus the amplitude ratio (Supplementary Note 1)^20^,^32^. The inhomogeneous intensity distribution in the phase shift image corresponds to position-dependent viscoelasticity in the glassy sample, originating from spatial heterogeneity caused by local structure variation^20^. Importantly, the phase shift in the images is independent of the surface roughness (Supplementary Figs 5 and 6). The colour range in the image is set according to the Gaussian distributions of phase shift angles (Supplementary Fig. 7). After structural relaxation at *T*_a_=553 K for 5 min, the characteristic length of the spatial heterogeneity becomes smaller, accompanying with a more dispersed distribution of high phase shift regions in the matrix (Fig. 3b). For a longer annealing duration of 720 min at *T*_a_=553 K, the β-relaxation leads to a further decrease in size of the domains which correspond to the spatial heterogeneity (Fig. 3c). The correlation function *P*(*r*)=2*σ*^2^[1−exp(−(*r*/*ξ*)^2^^*α*^)] is applied to quantitatively evaluate the characteristic length of the spatial heterogeneity (Fig. 3d), where *σ* is the standard deviation of phase shift values, *α* is the exponent and *ξ* is the lateral correlation length and defines the characteristic lengths between two correlated points in the phase shift images^20^,^32^. The correlation length of the spatial heterogeneity in the hyper-quenched metallic glass is measured to be ∼6.13 nm. Although this value appears to be much smaller than the sizes of the prominent features of phase shift image, the zoom-in AM-AFM micrograph verifies that the correlation length represents the mean sizes of spatial heterogeneity in local regions over the entire image (Supplementary Fig. 8). After annealing for 5, 30, 180 and 720 min at *T*_a_=553 K, the correlation length sequentially decreases to around 5.05, 4.28, 3.67 and 2.59 nm. The samples annealed at lower *T*_a_ of 473, 503 and 523 K show slower processes of the correlation length shrinkage (Supplementary Figs 9 and 10). From the phase shift and actual amplitude images, the distribution of dissipation energy can be obtained, which directly reflects the heterogeneity of structure and mechanical properties of the glass samples^20^. After sub-*T*_g_ relaxation, the amplitudes of the dissipation energy *E*_dis._ become smaller and the distribution profiles of *E*_dis._ turn out to be sharper (Supplementary Fig. 11), implying that the structure of the metallic glass develops more uniform with the disappearance and shrinkage of the high-energy-dissipated domains.

### Dynamics of heterogeneity evolution

**Figure 4:**
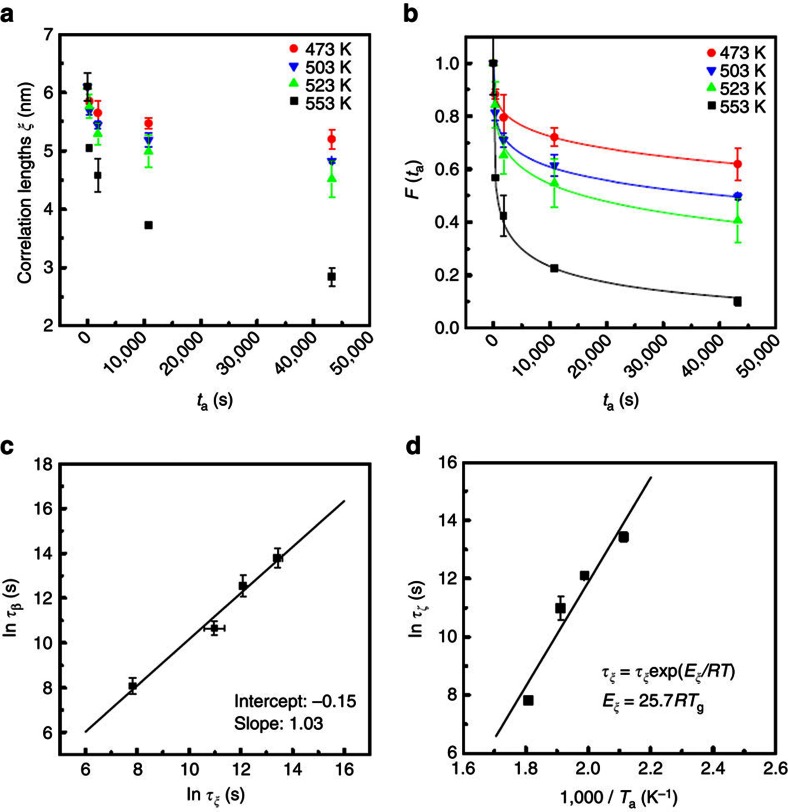
Dynamics of the nanoscale spatial heterogeneity. (**a**) The evolution of the correlation lengths *ξ* of the samples relaxed at 473 K, 503 K, 523 K and 553 K for different durations. (**b**) Annealing duration *t*_a_ dependences of the normalized correlation length cubed *F*(*t*_a_). (**c**) A comparison between the characteristic relaxation times *τ*_β_ and *τ*_ξ_ derived from the sub-*T*_g_ enthalpy relaxation and the evolution of spatial heterogeneity, respectively. (**d**) The activation energy of the evolution of spatial heterogeneity derived from the dependence of characteristic relaxation time *τ*_ξ_ of the spatial heterogeneity volumes on the reciprocal annealing temperature (1/*T*_a_). The error bars indicate standard deviation.

We analyse the dynamics of the spatial heterogeneity evolution during the sub-*T*_g_ relaxation at 473, 503, 523 and 553 K. The mean values of correlation lengths are plotted with *t*_a_ for all samples in Fig. 4a, showing a *T*_a_-dependent relaxation behaviour. We use the KWW function 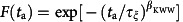 to describe the relaxation behaviour of the spatial heterogeneity, where *τ*_*ξ*_ is the characteristic relaxation time for the evolution of spatial heterogeneity. Since the spatial heterogeneity evolves in a three-dimensional space, the normalized correlation length cubed, *F*(*t*_a_)=(*ξ/ξ*_0_)^3^, where *ξ*_0_ is the correlation length of the hyper-quenched metallic glass, is used to fit the structure evolution at each annealing temperature. The nearly perfect fittings for the data acquired at four annealing temperatures (Fig. 4b) imply that the degeneration of the spatial heterogeneity is related to the sub-*T*_g_ dynamics of the metallic glass^1^,^17^. Similar to sub-*T*_g_ enthalpy release, the relaxation time *τ*_ξ_, derived from the KWW fitting, becomes shorter at higher *T*_a_. Importantly, as shown in Fig. 4c and Table 1, the relaxation time *τ*_ξ_ is approximately equal to the characteristic relaxation time *τ*_β_ of the sub-*T*_g_ enthalpy relaxation at the same annealing temperatures, indicating the inherent relationship between sub-*T*_g_ enthalpy release and the evolution of spatial heterogeneity. According to the Arrhenius equation, the activation energy *E*_ξ_ for the structural evolution is given to be (25.7±5.2)*RT*_g_ (Fig. 4d). Again, this value is close to the activation energy *E*_β_ for β-relaxation^4^,^6^,^7^,^16^,^34^,^35^. The relatively large variation of the measured activation energy values for both enthalpy release and spatial heterogeneity evolution is mainly due to the experimental error. Especially, the high-temperature data points (553 K) tend to form a curve in the linear Arrhenius plots (Fig. 1d and Fig. 4d), indicating a higher activation energy required for the high-temperature structure relaxation. As shown in Supplementary Fig. 1d, one can see that after annealing at 553 K for 720 min, the heat flow trace above the glass transition temperature has a slight deviation from the hyper-quenched one, suggesting that insignificant α-relaxation, which requires a much higher activation energy than β-relaxation, may be involved into the long-term sub-*T*_g_ relaxation and leads to the deviation of the 553 K data points from the linear Arrhenius plots.

## Discussion

The equivalences in relaxation time and activation energy between spatial heterogeneity evolution and sub-*T*_g_ enthalpy relaxation suggest that the underlying physical processes for the two different phenomena are dynamically identical. In fact, the dynamic-structure correspondence also unveils the micromechanisms of β-relaxation. Apparently, the β-relaxation cannot be attributed to all the atoms in mechanically rigid metallic glasses because a permanent macroscopic deformation has not been observed during mechanical or thermal stimulations at β-relaxation timescale. Thus, one reasonable scenario for β-relaxation in metallic glasses is local atomic motions or short-range diffusion confined to isolated small regions, which are energetically unstable and give rise to high energy dissipation during AM-AFM scanning and enthalpy release during thermal relaxation. The evolution of spatial heterogeneity with β-relaxation provides direct evidence on the scenario, that is, the β-relaxation is realized by very local structure changes, rather than global atomic motion. On the other hand, the local atomic re-arrangement through short-range diffusion or cooperative atomic motion^14^,^16^, driven by the release of the excess enthalpy, leads to the structure evolution of the hyper-quenched metallic glass and thereby the degeneration of the spatial heterogeneity.

The origins of the spatial heterogeneity in glasses have not been completely understood^17^. Apparently, it inherits from low-temperature supercooled liquids and represents the frozen state of supercooled liquids during glass transition^36^. Therefore, the spatial heterogeneity could be the result of heterogeneous dynamics of supercooled liquids, associated with density fluctuations^11^,^37^, frustration-limited domains^38^,^39^ or the structural basis of fragility^40^. It may also be created as relics of the liquid–liquid transition when the cooling rate is high enough, such as hyper-quenching, to completely freeze the liquid structure^41^. As revealed by Angstrom-beam electron diffraction^42^, the local atomic dense packing in metallic glasses, such as icosahedra, experiences geometric frustration because the free-energy-preferred atomic configurations cannot fill space efficiently and fails to extend indefinitely, giving birth to the frustration-limited domains or the spatial heterogeneity in glasses. As evidenced by the local energy dissipation and enthalpy release, the spatial heterogeneity observed in this study is related to the formation of a high-energy ‘phase' in comparison with the relatively stable matrix. Therefore, after the sub-*T*_g_ annealing, the strength of β-relaxation gradually decreases, together with the degeneration of the high-energy-dissipated domains. This structure change may lead to the loss of active sites that act as shear transformation zones to respond to the applied external forces for plastic deformation^10^,^25^,^28^,^43^,^44^ and thereby results in the annealing-induced embrittlement^45^, one of the major challenges for commercialization of metallic glasses^46^. The intrinsic correlation between spatial heterogeneity and β-relaxation revealed by this study may also explain the rejuvenation^47^ and temperature-dependent fast secondary relaxation^48^ of metallic glasses by non-affine thermal strains. The mismatch in local thermal expansion caused by spatial heterogeneity can lead to increased geometric frustration and high-energy domains with the growth of spatial heterogeneity at low temperatures and during the thermal cycling.

In summary, we report the evolution of spatial heterogeneity in a hyper-quenched metallic glass during sub-*T*_g_ β-relaxation. The characteristic relaxation times and activation energy of the spatial heterogeneity dynamics are in well accordance with those of β-relaxation, evidencing the intrinsic correlation between local structure evolution and sub-*T*_g_ β-relaxation. The microscopic connection between structure and dynamics of metallic glasses provides compelling evidence on the structural origin of β-relaxation and has important implications in understanding the mechanical properties and dynamics of metallic glasses.

## Methods

### Sample preparation

A hyper-quenched metallic glass with a composition of Zr_53_Cu_36_Al_11_ (atomic %) is prepared using RF magnetron sputtering with a deposition rate of∼0.2 nm s^-1^ at room temperature^49^. The thickness of the glass films on silicon (100) substrates is about 4 μm. By carefully controlling the deposition conditions, a mirror-like smooth surface with a sub-nanoscale roughness are achieved from the as-deposited films. The nominal cooling rate is evaluated to be ∼2.4 × 10^7^ K s^−1^ according to the scaled Arrhenius plot of fictive temperatures versus cooling rates^16^.

### Sub-*T*_g_ relaxation

The sub-*T*_g_ relaxation is performed using the hyper-quenching–annealing–calorimetric scan approach^7^. The samples are annealed in a Pt furnace with flowing pure Ar gas to prevent possible surface oxidation. The thermal properties of the glass are measured using DSC (Perkin-Elmer 8500). The heating and cooling rates for thermal scanning are set to be 20 K min^−1^. The standard sample is prepared by slowly cooling the hyper-quenched sample from the supercooled liquid region at 20 K min^−1^ and then subjected to the second up-scan to obtain the standard heat flow trace.

### Amplitude-modulation AFM

The hyper-quenched and relaxed samples are directly used for the AM-AFM testing without any polishing or surface processing to avoid damage and contamination to the sub-nanoscale smooth surfaces. The AM-AFM measurements are performed by a scanning probe microscope (Bruker MultiMode) at a tapping mode with a Nanoscope V controller. A pyramidal silicon tip with a sharp diamond-like spike of∼1 nm and spring constant around 5 N m^-1^ is driven vibrating near the resonant frequency (∼ 160 kHz) of the Si cantilever to scan across the top surfaces of samples. Surface height, phase shift and amplitude images are recorded simultaneously during the scanning. At least three different locations are scanned for each sample to get the mean values. The amplitude ratio is set to be around 0.85 during the scanning to avoid the damage of the sharp spike.

### Electron microscopy

Transmission electron microscopy samples are carefully prepared by the ion milling with 3 keV Ar ions at the liquid nitrogen temperature. High-resolution TEM and high-angle annular dark-field scanning TEM observations are conducted using a Cs-corrected TEM (JEM-2100 F, JEOL) with double spherical aberration (Cs) correctors for both the probe-forming and image-forming objective lenses.

## Additional information

**How to cite this article:** Zhu, F. *et al*. Intrinsic correlation between β-relaxation and spatial heterogeneity in a metallic glass. *Nat. Commun.* 7:11516 doi: 10.1038/ncomms11516 (2016).

## Supplementary Material

Supplementary Information Supplementary Figures 1-12, Supplementary Note 1 and Supplementary References

## Figures and Tables

**Table 1:** The characteristic relaxation times ***τ*_ξ_** and ***τ*_β_**.

***T***_**a**_ **(K)**	**ln** ***τ***_**ξ**_ **(s)**	**ln** ***τ***_**β**_ **(s)**
473	13.43±0.16	13.80±0.42
503	12.16±0.12	12.56±0.49
523	10.98±0.40	10.66±0.32
553	7.82±0.03	8.08±0.37
