# Supplementary material for: Intrinsic correlation between β-relaxation and spatial heterogeneity in a metallic glass
Source: Nat Commun. 2016 May 9;7:11516. doi: 10.1038/ncomms11516 (PMC4865810; doi:10.1038/ncomms11516)
Supplement: Supplementary Information — Supplementary Figures 1-12, Supplementary Note 1 and Supplementary References [file ncomms11516-s1.pdf]

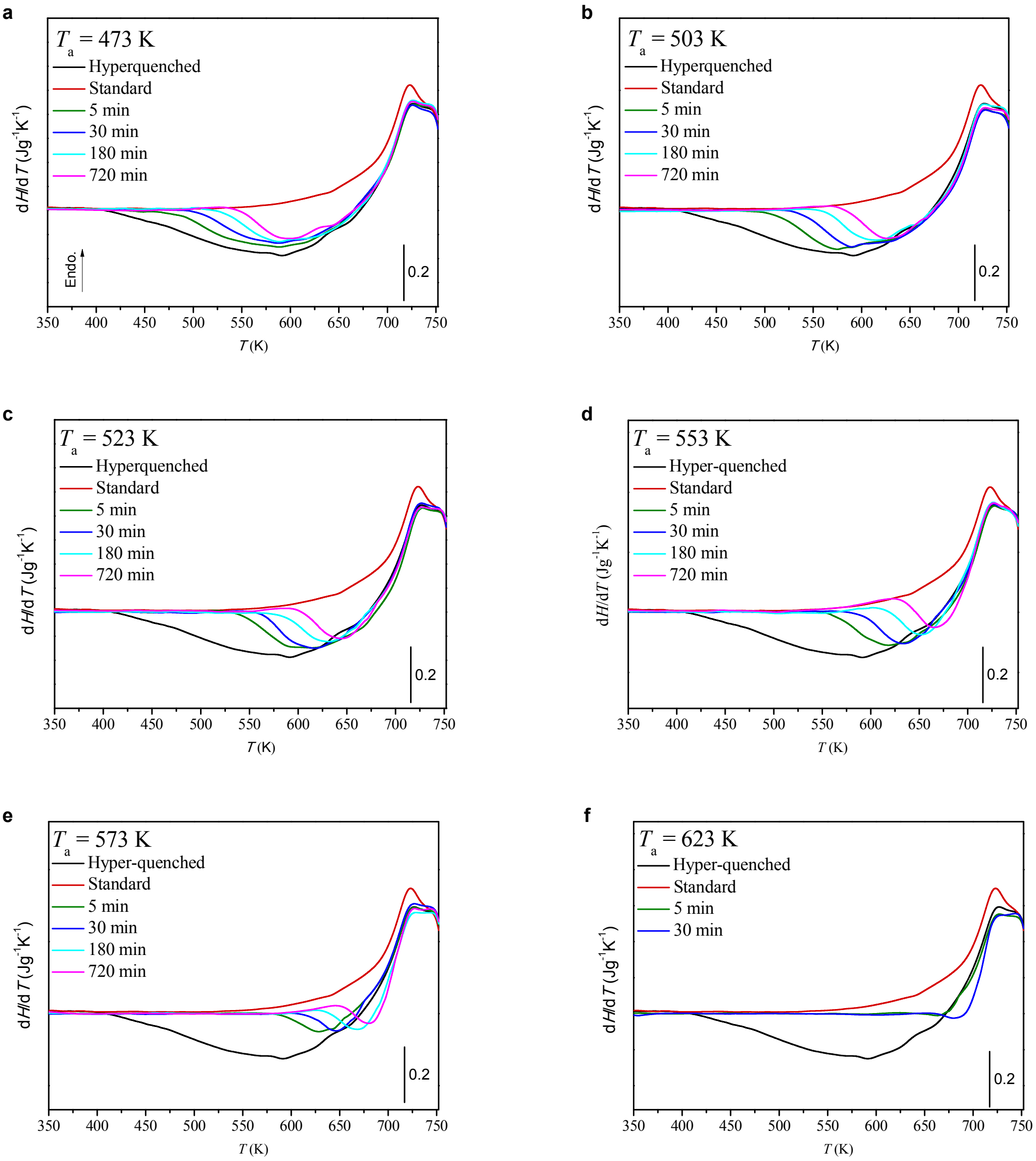

Supplementary Figure 1 | Heat flow traces of sub-T_g enthalpy relaxation. **(a)** Annealing tempeature T_a = 473 K (0.68T_g); **(b)** 503 K (0.72T_g); **(c)** 523 K (0.75T_g); **(d)** 553 K (0.8T_g); **(e)** 573 K (0.82T_g) and **(f)** 623 K (0.9T_g) for differrent durations. When the enthaply relaxation temperatures are above 0.8T_g, the α-relaxation or the glass transition will intervene if the annealing time is longer than 180 min for T_a = 573 K or 30 min for T_a = 623 K.

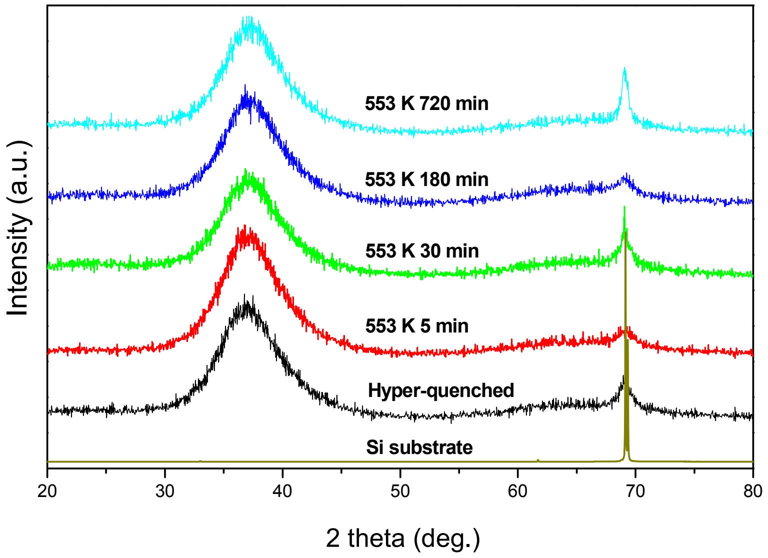

Supplementary Figure 2 | XRD patterns of the samples annealed at 553 K. The sharp peaks overlapping with the second diffraction halos come from the Si substrate.

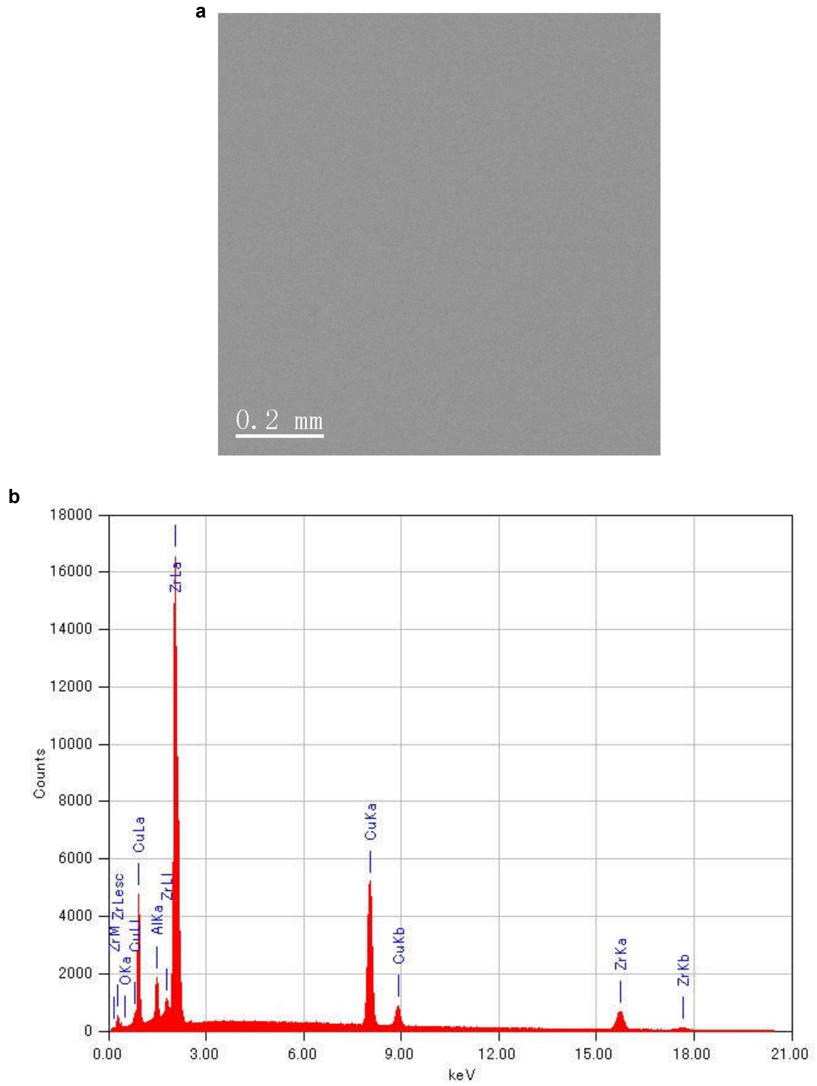

Supplementary Figure 3 | Metallic glass samples after long-time annealing at 553 K for 720 min. **(a)** SEM image of the sample surface showing no contamination introduced by the annealing. **(b)** Energy dispersive X-ray spectrum of the samples indicating that no obvious oxidation occurs during the annealing.

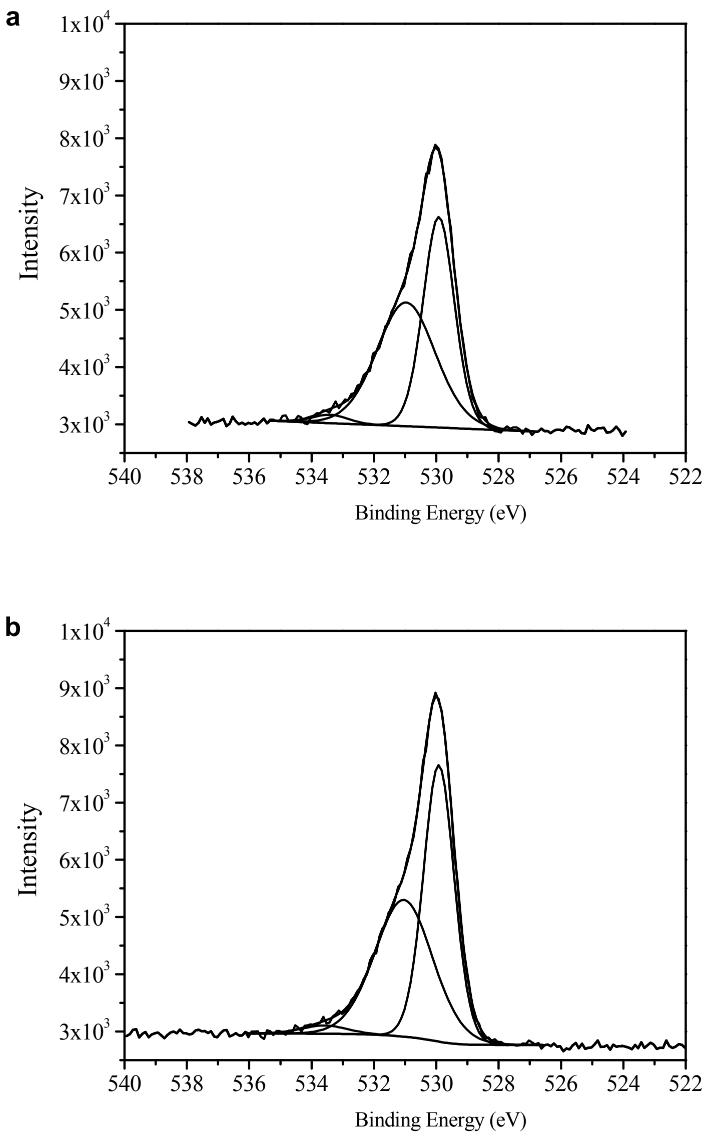

Supplementary Figure 4 | The XPS O1s spectra of samples. **(a)** The hyper-quenched metallic glass and **(b)** the sample annealed at 553 K for 720 min.

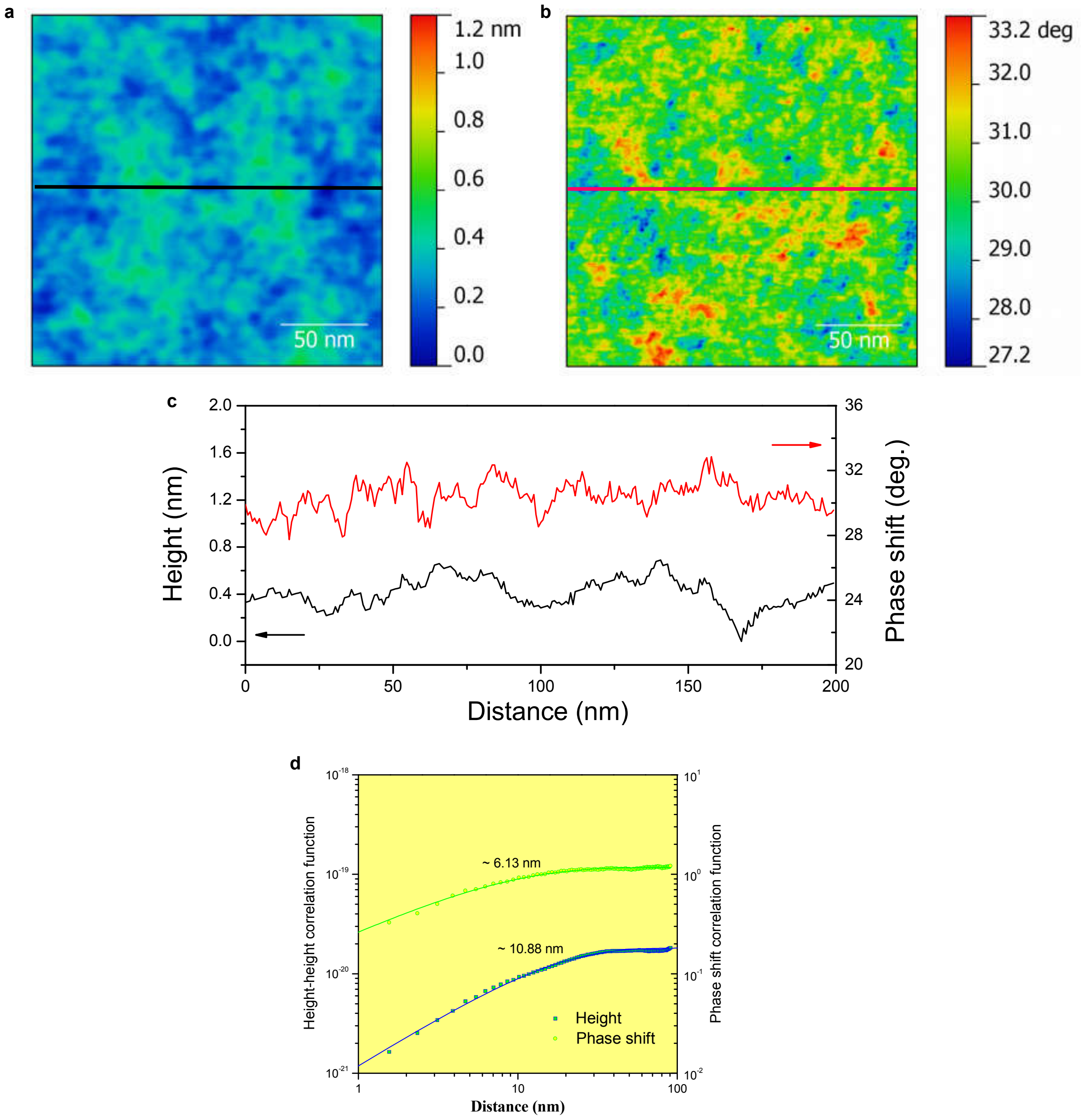

Supplementary Figure 5 | AM-AFM results of the hyper-quenched metallic glass. (a) Height image. **(b)** Phase shift image. **(c)** Profiles taken along the same line indicating that the phase shift is not correlated with the surface roughness. (d) Height-Height correlation function curves of the height and phase shift images.

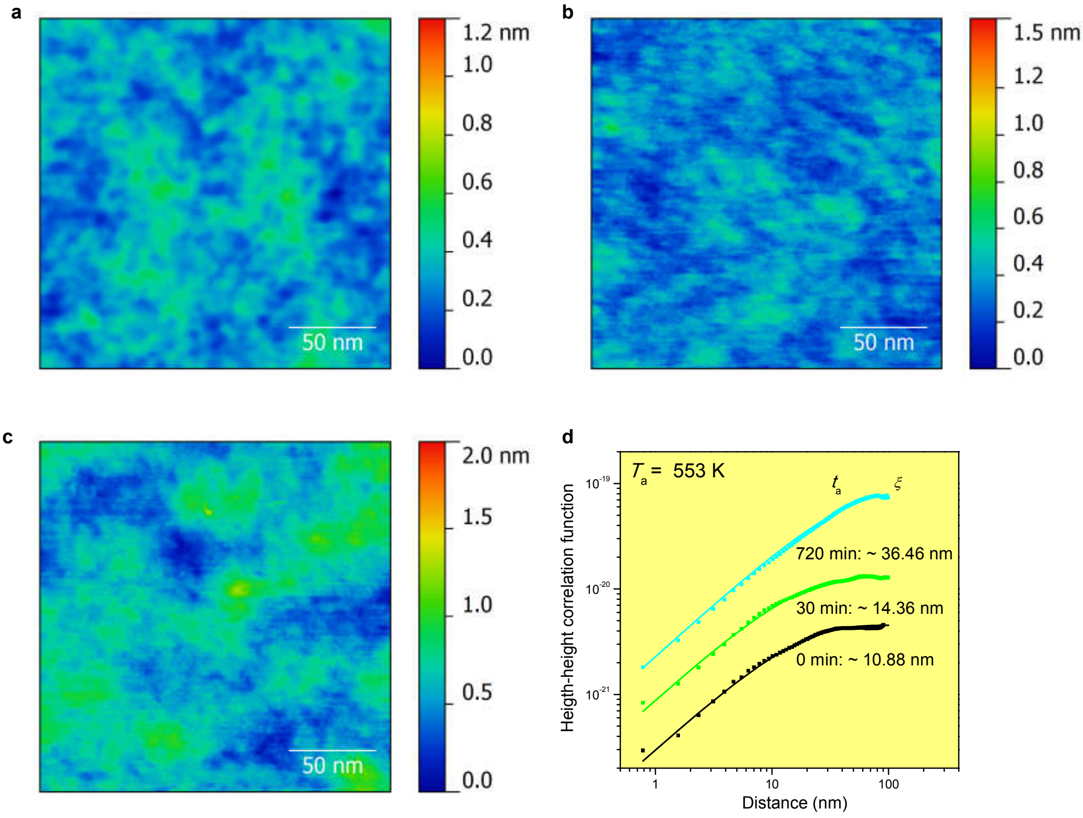

Supplementary Figure 6 | AM-AFM height images of samples before and after sub-T_g relaxation. **(a)** Hyper-quenched metallic glass. **(b)** The sample annealed at 553 K for 30 min. **(c)** The sample annealed at 553 K for 720 min. **(d)** The correlation function curves of the height images. The curves were shifted vertically for clear identification. The correlation lengths of the height images are 14.36 and 36.46 nm for the sample annealed for 30 min and 720 min, which are much larger than the correlation lengths of phase shift images.

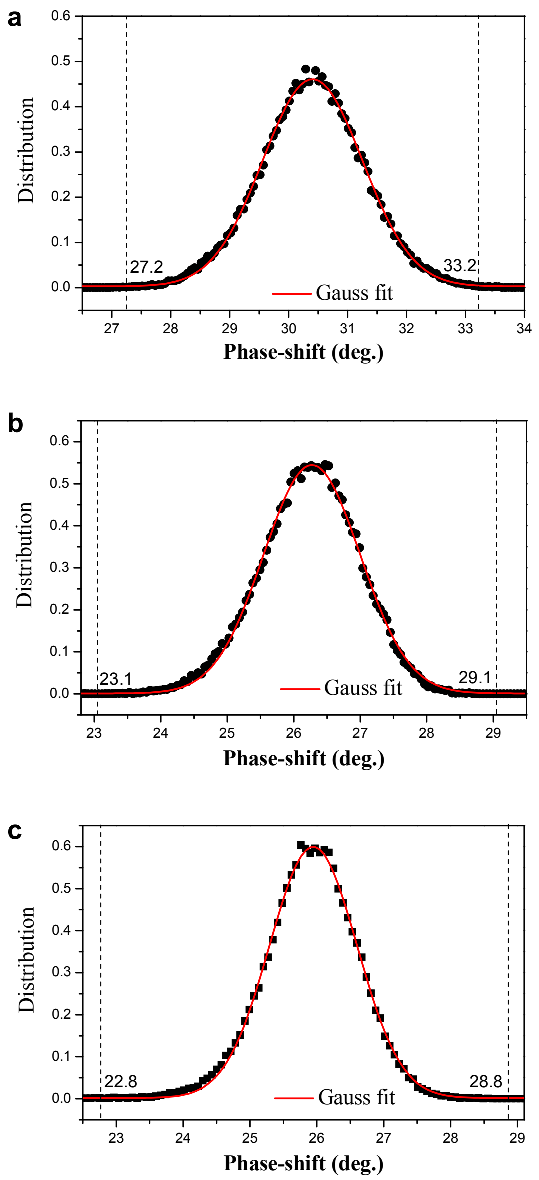

Supplementary Figure 7 | The statistic distributions of phase shift angles and the fitting by Gaussian functions. **(a)** Hyper-quenched metallic glass, and the samples annealed at 553 K for **(b)** 5 min; and **(c)** 720 min.

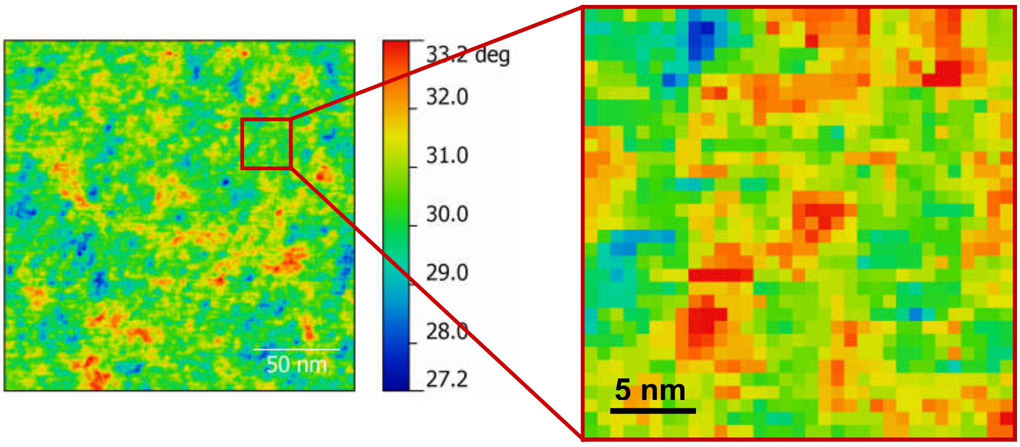

Supplementary Figure 8 | Zoom-in phase shift image of the hyper-quenched metallic glass. The feature length of local spatial heterogeneity is around 5-7 nm, which is in accord with the correlation length around 6.13 nm derived from the correlation function.

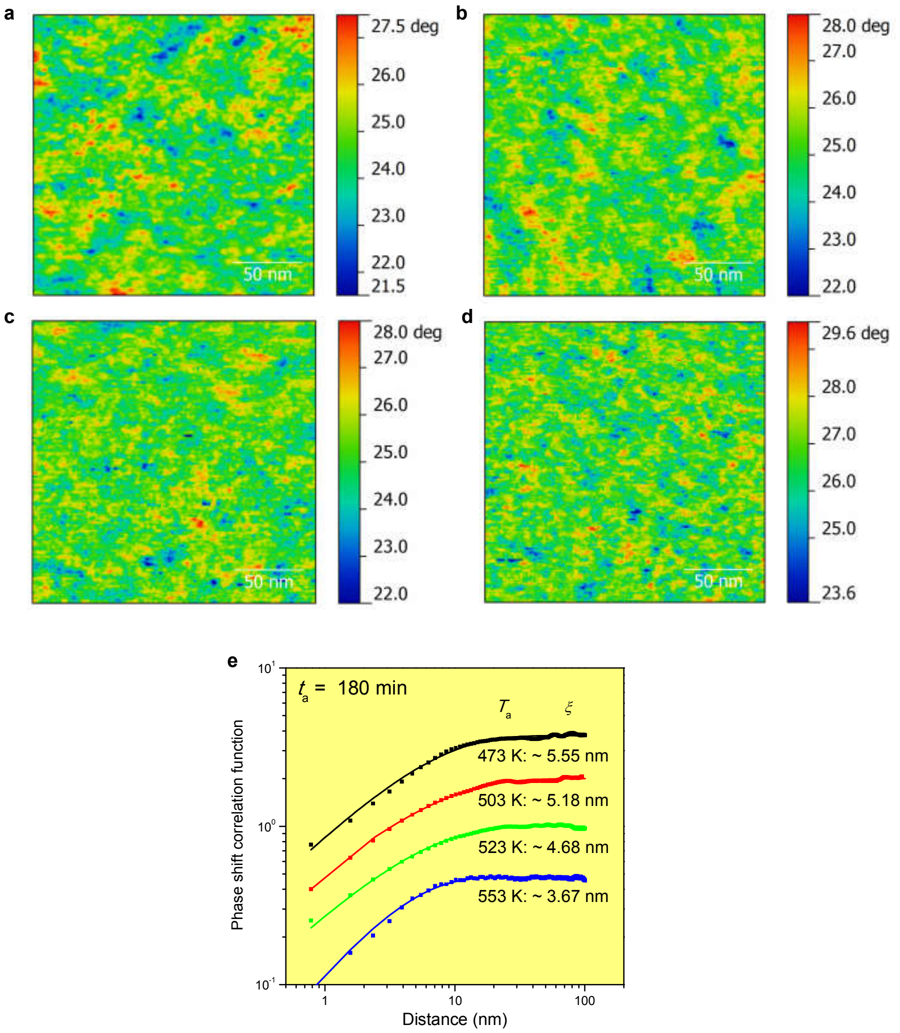

Supplementary Figure 9 | Evolution of nanoscale spatial heterogeneity during sub-T_g relaxation for t_a = 180 min. **(a)** T_a = 473 K; **(b)** T_a = 503 K, **(c)** T_a = 523 K and **(d)** T_a = 553 K. **(e)** The corresponding correlation functions. Note that the curves were shifted vertically for clear identification.

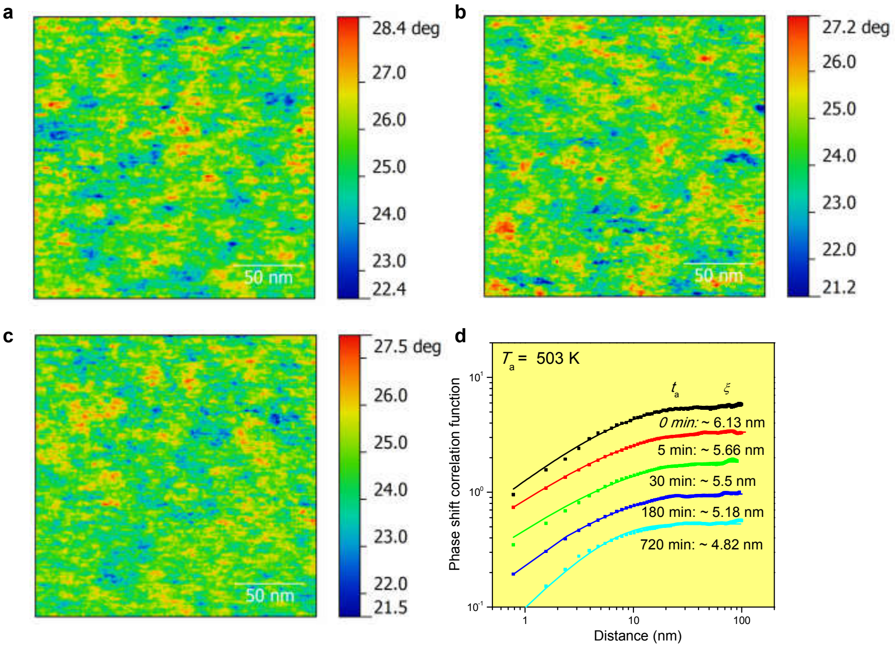

Supplementary Figure 10 | Evolution of spatial heterogeneity during sub-T_g relaxation at T_a = 503 K. Phase shift images of the samples annealed for (a) 5 min, (b) 30 min and (c) 720 min. (d) Correlation function curves of the samples annealed at 503 K for different durations. The correlation lengths of the spatial heterogeneity in the phase shift images were determined by the correlation function curves. Note that the curves were shifted vertically for clear identification.

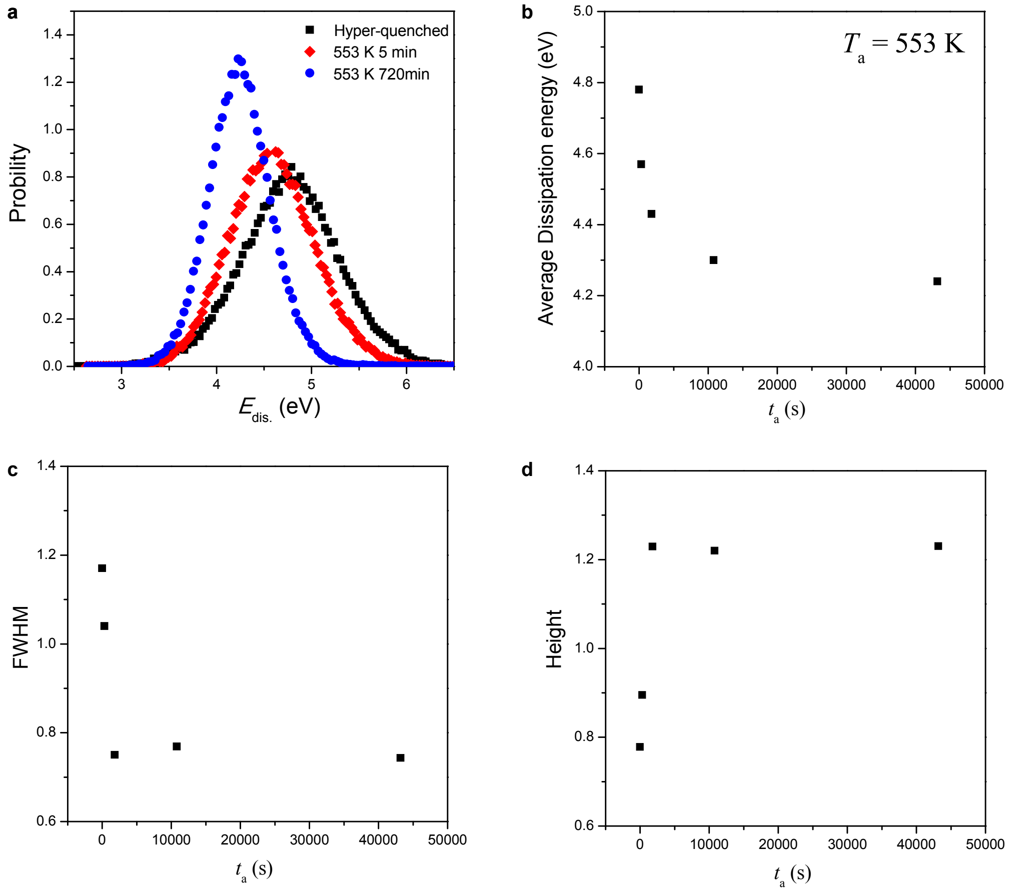

Supplementary Figure 11 | (a) The distributions of dissipation energy of the hyper-quenched metallic glass after sub-T_g relaxation at 553 K. **(b)** The mean values of dissipation energy; **(c)** the full width at half magnitude of the distributions; and **(d)** the height for the distribution plotted with the annealing time.

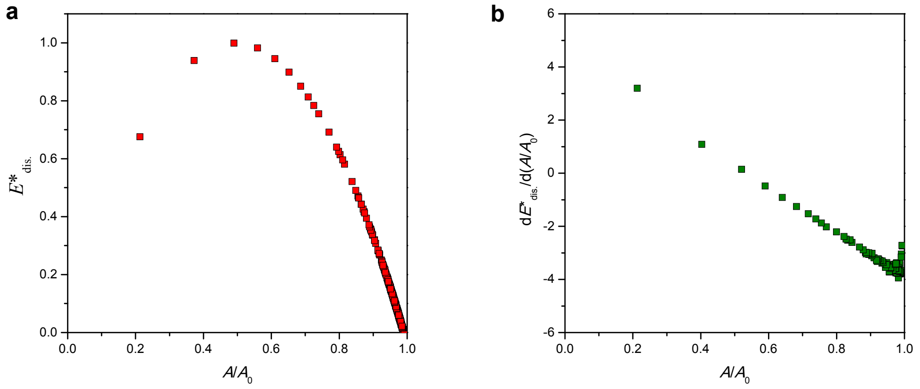

Supplementary Figure 12 | (a) The dynamic-dissipation curve, and **(b)** its derivative as function of the amplitude ratio A/A_0 (For details, see Supplementary Note 1).

Supplementary Notes

Supplementary Note 1

Energy dissipation mechanism. It has been demonstrated that viscoelasticity and surface energy hysteresis are two possible origins of the measured phase shift in metallic glasses [R1, R2]. Their contributions can be readily discriminated by measuring the normalized energy dissipation $E^{*}_{dis.}$ vs. A/A_0 curve, where $E^{*}_{dis.}=E_{dis.}/E^{max}_{dis.}$, with $E^{max}_{dis.}$ the maximum of the $E_{dis.}$, and A/A_0 is the amplitude ratio. According to the tip-sample interaction, either viscoelasticity or surface energy hysteresis has its own unique features in the $E^{*}_{dis.}$ vs. A/A_0 and $\delta E^{*}_{dis.}/\delta(A/A_0)$ vs. A/A_0 curves. For materials exhibiting viscoelastic behavior, the tip-sample interaction relies on both deformation and the deformation rate, giving rise to the dissipation inflection at the end of the A/A_0 range, and thereby the $\delta E^{*}_{dis.}/\delta(A/A_0)$ vs. A/A_0 curve is featured by an extreme point. We plotted the average $E^{*}_{dis.}$ vs. A/A_0 and $\delta E^{*}_{dis.}/\delta(A/A_0)$ vs. A/A_0 curves of the metallic glass (supplementary Fig. 12), which exhibit the typical characteristics of viscoelasticity of metallic glasses (Y. H. Liu, et al. *PRL* 106, 125504 (2011)). Particularly, in the derivative curve (Fig. S12b) we can see an extreme point at the end of the A/A_0 range, a feature of viscoelasticity. Therefore, the phase shift and thereby the energy dissipation, observed by AM-AFM in this study, mainly originate from the viscoelastic behavior of the metallic glass.

Supplementary References

1. Garcia, R., Gomez, C. J., Martinez, N. F., Patil, S., Dietz, C., & Magerle, R. Identification of nanoscale dissipation processes by dynamic atomic force microscopy. *Phys. Rev. Lett.* **97**, 016103 (2006).
2. Liu, Y. H., Wang, D., Nakajima, K., Zhang, W., Hirata, A., Nishi, T., Inoue, A. & Chen, M. W. Characterization of nanoscale mechanical heterogeneity in a metallic glass by dynamic force microscopy. *Phys. Rev. Lett.* **106**, 125504 (2011).
